# Anaplasmataceae closely related to *Ehrlichia chaffeensis* and *Neorickettsia helminthoeca* from birds in Central Europe, Hungary

**DOI:** 10.1007/s10482-020-01415-4

**Published:** 2020-04-21

**Authors:** Sándor Hornok, Sándor A. Boldogh, Nóra Takács, Alexandra Juhász, Jenő Kontschán, Dorottya Földi, Balázs Koleszár, Pál Morandini, Miklós Gyuranecz, Sándor Szekeres

**Affiliations:** 1 Department of Parasitology and Zoology, University of Veterinary Medicine, Budapest, 1078 Hungary; 2 Department of Nature Conservation, Aggtelek National Park Directorate, Jósvafő, 3758 Hungary; 3 Department of Zoology, Plant Protection Institute, Centre for Agricultural Research, Budapest, 1022 Hungary; 4 Zoonotic Bacteriology and Mycoplasmatology Research Team, Institute for Veterinary Medical Research, Centre for Agricultural Research, Budapest, 1143 Hungary; 5 Hungarian Ornithological and Nature Conservation Society, Budapest, 1125 Hungary

**Keywords:** Vector-borne, Tick-borne, Rickettsia, Piroplasm, Borrelia, *Francisella*, *Coxiella*

## Abstract

Increasing amount of data attest that (in the context of vector-borne infections) birds are not only important as hosts of blood-sucking arthropod vectors, but also as reservoirs of vector-borne pathogens. From 2015 to 2019 cadavers of 100 birds (from 45 species, nine orders) were collected in Hungary, and their organs were screened for DNA from a broad range of vector-borne bacteria with PCR and sequencing. Molecular analyses revealed the presence of Anaplasmataceae, and sequencing identified bacteria closely related to *Neorickettsia helminthoeca* and *Ehrlichia chaffeensis* in a Eurasian teal (*Anas crecca*) and a song thrush (*Turdus philomelos*), respectively. All samples were PCR negative for rickettsiae, borreliae, *Francisella* and *Coxiella* spp., as well as for piroplasms. To our knowledge, this is the first report of a *Neorickettsia* and an *Ehrlichia* sp., which belong to the phylogenetic groups of *N. helminthoeca* and *E. chaffeensis*, respectively, from Europe. The potential presence of these two vector-borne bacteria needs to be taken into account during future studies on the eco-epidemiology of Anaplasmataceae in Europe.

**Electronic supplementary material:**

The online version of this article (10.1007/s10482-020-01415-4) contains supplementary material, which is available to authorized users.

## Introduction

Vector-borne microorganisms are those, which can be transmitted by blood-sucking arthropods or other invertebrate vectors between susceptible vertebrate hosts (Vaughan et al. 2012). In several regions of the globe even molecular “baseline data” are lacking on the occurrence of vector-borne pathogens in various vertebrate groups, therefore the emerging or receding character of these infections might be hard to assess. From an epidemiological point of view, birds are perhaps the most important to study in this context, because of their motility (migration habit) and frequent association with arthropod vectors, such as ticks (de la Fuente et al. 2015).

Birds are often reported as hosts of ticks and other blood-sucking arthropods, which may transmit vector-borne pathogens of veterinary-medical importance (de la Fuente et al. 2015; Farajollahi et al. 2011). However, compared to mammals, the reservoir role of birds appears to have deserved less attention from the point of view of vector-borne pathogens, as exemplified by the limited number of studies on borreliae (Ginsberg et al. 2005), rickettsiae (Hornok et al. 2014) and Anaplasmataceae (Keesing et al. 2012). Therefore, this study aimed at screening and identifying vector-borne bacteria and protozoa (rickettsiae, borreliae, *Francisella* and *Coxiella* spp., as well as piroplasms) in birds from Central Europe, Hungary. The results focus on Anaplasmataceae.

## Materials and methods

One hundred bird cadavers (belonging to 45 species, nine orders: Supplementary file 1) were collected between January 2015 and January 2019, at three locations in north (Budapest, n = 18) and north-eastern Hungary (Aggtelek National Park and its surroundings, n = 40; Eger, n = 13), as well as in western Hungary (Sopron, n = 7) and other parts of the country (in total n = 22; in particular: Bugyi, n = 1; Dinnyés, n = 1; Gárdony, n = 1; Hajdúböszörmény, n = 1; Hajdúszoboszló, n = 1, Zánka, n = 2; not recorded, n = 15). All birds were found dead due to natural causes or car hits, therefore no ethical permission was needed. Utilization of cadavers for scientific purposes was in accordance with the government decree 71/2015.[III.30.]. From the cadavers at least 200 μl of EDTA-anticoagulated blood was collected, if possible. In addition, 100 mg tissue samples were taken from the middle of organs which were in good condition (i.e. intact, not autolyzed), with sterile scalpel blade to exclude surface contamination. Tissues sampled for DNA extraction included the spleen, liver and heart muscle (Supplementary file 1), but other organs (lungs, kidneys, brain) were also preserved. All tissues were kept frozen at − 20 °C until processing. DNA was extracted with the QIAamp DNA Mini Kit (Qiagen, Hilden, Germany) following the manufacturer’s instruction and including extraction control (100 μl phosphate-buffered saline processed together with the samples) in each set of samples to monitor cross contamination. All endo- or ectoparasites were collected and stored in 96% ethanol.

In total, 273 DNA extracts and 12 extraction controls were screened for a broad range of vector-borne bacteria and piroplasms (Supplementary file 2). Investigations for the presence of Anaplasmataceae were performed in two steps (Table 1). The first step was a screening assay, which was followed by group-specific PCRs, amplifying a short or long part of the 16S rRNA gene, respectively. This gene was chosen as the target of molecular-phylogenetic analyses, on account of its suitability for screening, as well as for genotyping *Neorickettsia* and *Ehrlichia* species (Vaughan et al. 2012; Rar and Golovljova 2011). All PCRs included appropriate positive and negative (non-template) controls. Positive controls for Anaplasmataceae were sequence-verified *Anaplasma marginale* from cattle blood; *Ehrlichia* sp. HG-T10 from tick and *Neorickettsia* sp. AVTI-128 from bird. For other vector-borne pathogens positive controls are listed in Supplementary file 2.

**Table 1:** Data of 16S rRNA conventional PCRs used for screening Anaplasmataceae and sequencing *Neorickettsia* and *Ehrlichia* genotypes

Target group	Gene (~ amplicon length)	Oligonucleotides (5′—3′) (Reference)	Temperature and duration of:					Number of cycles
			Initial denaturation	Denaturation	Annealing	Extension	Final extension	
Anaplasmataceae	16S rRNA gene (350 bp)	EHR-16sD (GGT ACC YAC AGA AGA AGT CC) EHR-16sR (TAG CAC TCA TCG TTT ACA GC) (Brown et al. 2001)	95 °C, 10 m	95 °C, 30 s	55 °C, 30 s	72 °C, 45 s	72 °C, 5 m	40
*Neorickettsia* spp.	16S rRNA gene (1260 bp)	n16S-50F (TAG GCT TAA CAC ATG CAA GTC GAA CG)	95 °C, 5 m	95 °C, 40 s	56 °C, 30 s	72 °C, 1 m	72 °C, 5 m	40
		n16S-1400R (CGG TTA GCT CAC TAG CTT CGA GTA A) (Greiman et al. 2014)						
*Ehrlichia* spp.	16S rRNA gene (1290 bp)	EE-1 (TCC TGG CTC AGA ACG AAC GCT GGC GGC)	95 °C, 5 m	95 °C, 30 s	69 °C, 30 s	72 °C, 45	72 °C	40
		EE-2mod* (AGT CAC TAA CCC AAC CTT AAA TGG CTG) (Pusterla et al. 2000) *this study						

**Fig. 1:**
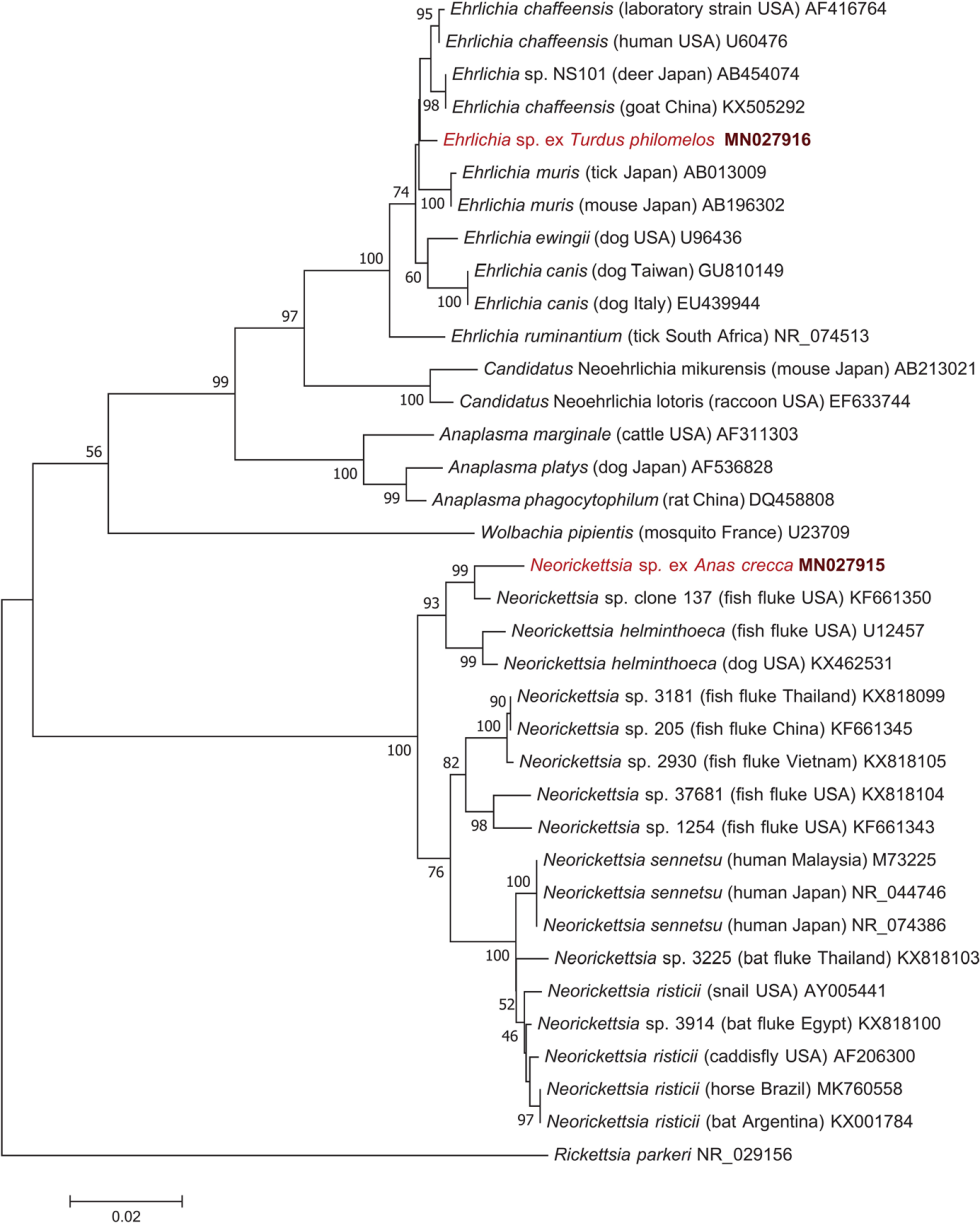
Minimum Evolution phylogenetic tree of Anaplasmataceae (with *Rickettsia parkeri* as outgroup) based on the 16S rRNA gene. There were a total of 1227 positions in the final dataset. Genotypes from this study are highlighted with red color and bold accession numbers. For each item, the isolation source and country of origin are shown in parentheses between the species name and relevant GenBank accession number. The scale-bar indicates the number of substitutions per site

Purification and sequencing were performed at Biomi Ltd. (Gödöllő, Hungary). Sequences were aligned and compared to GenBank sequences by nucleotide BLASTn program (https://blast.ncbi.nlm.nih.gov). Phylogenetic analyses were performed with the Minimum Evolution method (Tamura Nei model) (Fig. 1), the unweighted pair group method with arithmetic mean (UPGMA) (Supplementary file 3) and Maximum Likelihood (ML) method (Jukes-Cantor model) (Supplementary file 4). All sequences retrieved from GenBank for phylogenetic analysis had 99–100% coverage with ours. Phylogenetic analyses were done using MEGA 7.0 (Kumar et al. 2016).

## Results

All samples were PCR negative for rickettsiae, borreliae, *Francisella* and *Coxiella* spp., as well as for piroplasms. However, two samples were PCR positive for Anaplasmataceae, and were therefore analyzed further.

A new *Neorickettsia* genotype was detected in a Eurasian teal (*Anas crecca*: order Anseriformes). *Neorickettsia* DNA could be amplified from the blood sample, the kidneys, and the lungs. In this bird two fluke species were present: the blood fluke *Bilharziella polonica* (mounted on slide prior to knowing PCR-positivity of its host) and an *Echinostoma* sp. intestinal fluke (preserved in 96% ethanol, processed for DNA extraction, and PCR negative for Anaplasmataceae). Molecular analysis of the new *Neorickettsia* genotype revealed the highest, 97.7–98.9% (1224-1239/1253 bp) sequence identity with bacteria of the *N. helminthoeca* group reported from the USA (GenBank: U12457, KF661350 and KX462531). This was confirmed in the phylogenetic tree, because the new *Neorickettsia* genotype clustered together with *N. helminthoeca*-related isolates, and their separation from other neorickettsiae was strongly (100%) supported (Fig. 1).

An *Ehrlichia* genotype was present in a song thrush (*Turdus philomelos*: order Passeriformes). Only the blood and spleen samples of this bird were PCR positive, but its liver, heart muscle, kidneys, lungs, and brain were PCR negative. Molecular analysis of this *Ehrlichia* genotype showed the highest, 99.1–99.5% (1282-1287/1294 bp) sequence identity with *Ehrlichia chaffeensis*, reported from ruminants in China (KX505292) and Japan (AB454074) as well as humans in the USA (U60476). On the other hand, the *Ehrlichia* sp. from song thrush had lower, only 97.8–98.5% (1266-1275/1294 bp) sequence identity with *E. ruminantium* (NR_074513), *E. muris* (AB013009), *E. canis* (EU439944) and 98% (340/347 bp) identity with an *Ehrlichia* genotype recently reported from penguins (MK049840).

Phylogenetically, the avian *Ehrlichia* genotype reported here occupied a basal position to the group of American and/or Asian *Ehrlichia chaffeensis* isolates in the Minimum Evolution (Fig. 1), as well as in the UPGMA and ML trees (Supplementary files 3–4). Interestingly, this avian *Ehrlichia* genotype was 100% identical (690/690 bp) in its considerably shorter 16S rRNA gene sequence with *Ehrlichia* sp. "It20" and "It40" reported from ticks (*Ixodes turdus*) in Japan (GenBank: LC386012 and LC386013).

New sequences were submitted to GenBank (accession numbers: MN027915 and MN027916 for the *Neorickettsia* and *Ehrlichia* sp., respectively).

## Discussion

PCR-negativity of all analyzed specimens for rickettsiae, borreliae and piroplasms (for which birds are known to be susceptible: Hasle 2013) can be explained by either the lack of infection in examined birds, or very low levels of bacteraemia/parasitaemia not detectable by conventional PCRs used here. At the same time, even highly sensitive real-time PCRs were unable to show infection with *F. tularensis* and *C. burnetii* or closely related species in birds of this study, suggesting that the majority of analyzed bird species (e.g. songbirds: order Passeriformes, represented by 46 out of 100 individuals here) do not carry the causative agents of tularemia and Q-fever in relevant regions of Hungary. This is in line with literature data, because *F. tularensis* and *C. burnetii* often have the highest prevalence in diurnal birds of prey (Riemann et al. 1977; Padeshki et al. 2010), of which only 13 individuals (Supplementary file 1: order Accipitriformes) were included in the present study.

Bacteria transmitted to birds by arthropod *vs* fluke vectors may have different host-associations. Trushes (family Turdidae) are frequently reported to have the highest rate of tick infestation among songbirds (order Passeriformes) (Klaus et al. 2016), and this tendency may explain why the single PCR positivity for a genotype closely related to tick-borne ehrlichiae was detected in a song thrush here. On the other hand, flukes can be most often found in waterfowl and aquatic birds (Coles 2007), as exemplified by order Anseriformes in the present study. This order includes the Eurasian teal, in which a genotype closely related to fluke-associated neorickettsiae was detected.

Bacteria of the genus *Neorickettsia* are obligate intracellular endosymbionts of digenean flukes (Platyhelminthes: Digenea), from which they can also pass into vertebrates, inducing severe disease. Among the latter, salmon poisoning disease (SPD) is caused by *N. helminthoeca*. This species affects canids following ingestion of fluke-infected salmonid fish, and is known to occur in the western Pacific coast of North America, and in South America (Vaughan et al. 2012). To our knowledge, this is the first report of a *Neorickettsia* sp., belonging to the phylogenetic group of *N. helminthoeca*, from Europe. Neorickettsiae have been reported from at least six families of flukes in North America and Asia, which include species associated with (piscivorous) birds as final hosts (e.g. Lecithodendriidae, Schistosomatidae and Echinostomatidae: Vaughan et al. 2012), and these families are represented by several species in Hungary (Edelényi 1974). Nevertheless, in the present case the single *Echinostoma* specimen from the neorickettsia-positive bird turned out to be PCR negative and blood flukes could not be analyzed molecularly, leaving the fluke vector of this new neorickettsia genotype open to future studies. To our knowledge, this is also the first molecular evidence for a *Neorickettsia* species (closely related to *N. helminthoeca*) in the blood of any bird species.

Regarding other Anaplasmataceae, while there are several studies which demonstrated the presence of *Anaplasma phagocytophilum* in birds, particularly thrushes (Passeriformes: Turdidae) both in North America (Daniels et al. 2002) and in Europe (de la Fuente et al. 2005), there appear to be only two previous reports on the occurrence of *Ehrlichia* spp. in birds (order Falconiformes: Machado et al. 2012; order Sphenisciformes: Muñoz-Leal et al. 2019), both from South America. Unfortunately, both studies were based on short (approximately 350 bp long) 16S rRNA gene sequences, having only 30% coverage with ours, which therefore could not be included in the present phylogenetic analyses.

Taken together, this is the first finding of *Ehrlichia*-infection in birds in Europe, and in any passeriform bird species in a worldwide context. Moreover, the new *Ehrlichia* genotype detected in a songbird here is most closely related to *E. chaffeensis*, the causative agent of human monocytic ehrlichiosis with the highest number of cases in North America. Although seropositivity to *E. chaffeensis* was also recorded outside this main range (i.e., in southern Europe, Middle East and southern Asia), DNA was not detected in any of these cases (Rar and Golovljova 2011). In its shorter fragment of the 16S rRNA gene, the bird-associated *Ehrlichia* genotype detected here was most closely related to an *Ehrlichia* genotype reported from ticks (*Ixodes turdus*) in Japan (Taira et al. 2019). Interestingly, this tick species is associated with birds, particularly from the order Passeriformes (several families, including Turdidae), but it is only known to occur in the eastern Palaearctic far from Europe (Yamaguti et al. 1971). It is therefore an unlikely vector in the present context.

The new *Ehrlichia* genotype shown here to be present in a song thrush (*Turdus philomelos*) confirmed the potential epidemiological significance of thrushes as reservoirs of Anaplasmataceae from at least two genera: *Anaplasma* (as already known) and *Ehrlichia* (as shown here).

## Electronic supplementary material

Below is the link to the electronic supplementary material.

Supplementary file1 (PDF 63 kb)

Supplementary file2 (PDF 58 kb)

Supplementary file3 (PDF 326 kb)

Supplementary file4 (PDF 781 kb)

## Data Availability

The sequences obtained and/or analyzed during the current study are deposited in GenBank under accession numbers MN027915 and MN027916 for the *Neorickettsia* and *Ehrlichia* sp., respectively. All other relevant data are included in the manuscript and the references.
