# Supplementary material for: Anaplasmataceae closely related to Ehrlichia chaffeensis and Neorickettsia helminthoeca from birds in Central Europe, Hungary
Source: Antonie Van Leeuwenhoek. 2020 Apr 21;113(7):1067–73. doi: 10.1007/s10482-020-01415-4 (PMC7272389; doi:10.1007/s10482-020-01415-4)
Supplement: Supplementary file 1 — Supplementary file1 (PDF 63 kb) [file 10482_2020_1415_MOESM1_ESM.pdf]

Order	English name	Scientific name	n=	Sample number according to organ			
				Spleen	Heart	Blood	Liver
Galliformes	pheasant	*Phasianus colchicus*	1	1	1	1	1
Anseriformes	Eurasian teal	*Anas crecca*[1]	4	4	4	4	4
	mallard	*Anas platyrhynchos*	5	1	5	5	5
	greylag goose	*Anser anser*	7	1	4	4	4
Charadriiformes	dunlin	*Calidris alpina*	2	2	2	2	2
	common snipe	*Gallinago gallinago*	1	1	1	1	1
	Eurasian woodcock	*Scolopax rusticola*	1	-	1	-	1
Accipitriformes	northern goshawk	*Accipiter gentilis*	1	-	1	-	1
	northern sparrowhawk	*Accipiter nisus*	7	3	7	2	7
	common buzzard	*Buteo buteo*	4	4	4	1	4
	common kestrel	*Falco tinnunculus*	1	-	1	-	1
Strigiformes	long-eared owl	*Asio otus*	6	2	5	-	5
	little owl	*Athene noctua*	3	2	3	-	3
	Eurasian eagle-owl	*Bubo bubo*	1	-	1	1	1
	tawny owl	*Strix aluco*	1	1	1	1	1
	Ural owl	*Strix uralensis*	2	1	2	-	2
	barn owl	*Tyto alba*	2	-	2	-	2
Piciformes	middle spotted woodpecker	*Dendrocopos medius*	1	1	1	1	1
Columbiformes	rock pigeon	*Columba livia*	2	2	2	2	2
	common wood pigeon	*Columba palumbus*	1	-	1	-	1
	Eurasian collared dove	*Streptopelia decaocto*	1	-	1	-	1
Apodiformes	common swift	*Apus apus*	1	-	1	-	1

Passeriformes	sedge warbler	*Acrocephalus schoenobaenus*	1	-	1	1	1
	European goldfinch	*Carduelis carduelis*	1	-	1	-	1
	European greenfinch	*Carduelis chloris*	1	-	1	-	1
	hawfinch	*Coccothraustes coccothraustes*	5	1	5	-	5
	hooded crow	*Corvus corone cornix*	1	1	1	1	1
	yellowhammer	*Emberiza citrinella*	3	-	3	-	3
	European robin	*Erithacus rubecula*	6	4	5	1	5
	common chaffinch	*Fringilla coelebs*	1	-	1	-	1
	Eurasian jay	*Garrulus glandarius*	2	2	2	1	2
	lesser grey shrike	*Lanius minor*	1	1	1	-	1
	red-backed shrike	*Lanius collurio*	1	-	1	-	1
	white wagtail	*Motacilla alba*	1	-	1	-	1
	great tit	*Parus major*	2	1	2	-	2
	house sparrow	*Passer domesticus*	1	1	1	-	1
	Eurasian tree sparrow	*Passer montanus*	1	-	1	-	1
	black redstart	*Phoenicurus ochruros*	1	-	1	-	1
	Eurasian magpie	*Pica pica*	1	1	1	1	1
	goldcrest	*Regulus regulus*	1	-	1	-	1
	Eurasian blackcap	*Sylvia atricapilla*	1	-	1	-	1
	common blackbird	*Turdus merula*	5	4	4	2	5
	song thrush	*Turdus philomelos*[2]	5	1	5	1	5
	fieldfare	*Turdus pilaris*	2	2	2	1	2
	mistle thrush	*Turdus viscivorus*	2	2	2	1	2

[1]collected at Fertőújlak (Mekszikópuszta), August 2018

[2]collected at Eger, April 2018
