# Supplementary material for: Anaplasmataceae closely related to Ehrlichia chaffeensis and Neorickettsia helminthoeca from birds in Central Europe, Hungary
Source: Antonie Van Leeuwenhoek. 2020 Apr 21;113(7):1067–73. doi: 10.1007/s10482-020-01415-4 (PMC7272389; doi:10.1007/s10482-020-01415-4)
Supplement: Supplementary file 2 — Supplementary file2 (PDF 58 kb) [file 10482_2020_1415_MOESM2_ESM.pdf]

Technical Appendix 2. Technical data of conventional and real-time PCRs used for screening vector-borne pathogens other than Anaplasmataceae. In conventional PCRs, the reaction mixture (25 µl) contained 1 U (0.2 µl) HotStarTaq Plus DNA polymerase, 2.5 µl 10× CoralLoad Reaction buffer (including 15 mM $MgCl_2$), 0.5 µl PCR nucleotide Mix (0.2 mM each), 0.5 µl (1 µM final concentration) of each primer, 15.8 µl ddH_2O and 5 µl template DNA.

Target group	Gene (~ amplicon length)	Oligonucleotides (5' - 3') (Reference)	Temperature and duration of:					Number of cycles
			Initial denaturation	Denaturation	Annealing	Extension	Final extension	
Rickettsia spp.	gltA (380 bp)	RpCs.877p (GGG GGC CTG CTC ACG GCG G)<br>RpCs.1258n (ATT GCA AAA AGT ACA GTG AAC A)<br>(Regnery et al. 1991)	95°C, 5m	95°C, 20s	48°C, 30s	72°C, 1m	72°C, 5m	40
Borrelia burgdorferi s.l.	5S-23S IGS (420bp)	B5Sborseq (GAG TTC GCG GGA GAG TAG GTT ATT GCC)<br>23Sborseq (TCA GGG TAC TTA GAT GGT TCA CTT CC)<br>(Heylen et al. 2013)	94°C, 5m	94°C, 20s	70°C, 30s (-1°C/cycle)	72°C, 30s		10
				94°C, 20s	60°C, 30s	72°C, 30s	72°C, 7m	40
Borrelia miyamotoi	qlpQ gene (730bp)	qlpQ-BM-F2 (ATG GGT TCA AAC AAA AAG TCA CC)<br>qlpQ-BM-R1 (CCA GGG TCC AAT TCC ATC AGA ATA TTG TGC AAC)<br>(Hovius et al. 2013 / Szekeres et al. 2015)	95°C, 5m	94°C, 30s	62°C, 30s (-1°C/cycle)	72°C, 1m		10
				94°C, 30s	53°C, 30s	72°C, 1m	72°C, 10m	40
Coxiella spp.	IS1111 transposon	IS1111f (CCG ATC ATT TGG GCG CT)<br>IS1111r (CGG CGG TGT TTA GGC)<br>IS1111p (6FAM-TTA ACA CGC CAA GAA ACG TAT CGC TGT G-MGB)<br>(Loftis et al., 2006)	95°C, 10 min	95°C, 15s	60°C, 60s	-	-	45
Francisella spp.	tul4 (100 bp)	Tul4f (ATT ACA ATG GCA GGC TCC AGA)<br>Tul4r (TGC CCA AGT TTT ATC GTT CTT CT)<br>Tul4p (FAM-TTC TAA GTG CCA TGA TAC AAG CTT CCC AAT TAC TAA G-BHQ)<br>(Versage et al., 2003)	94°C, 10 min	94°C, 15s	60°C, 30s	72°C, 20s	-	45
piroplasms	18S rRNA (500 bp)	BJ1 (GTC TTG TAA TTG GAA TGA TGG)<br>BN2 (TAG TTT ATG GTT AGG ACT ACG)<br>(Casati et al. 2006)	95°C, 10m	95°C, 30s	54°C, 30s	72°C, 40s	72°C, 5m	40

Abbreviations: m=minutes, s=seconds.

Sequence-verified positive controls according to PCR target groups:

Rickettsia spp. - *R. helvetica* from tick

Borrelia burgdorferi s.l. - *B.afzelii*, *B.garinii*, *B. valaisiana* from tick

Borrelia miyamotoi - *B.miyamotoi* from tick

Coxiella spp. - *C. burnetii* strain Nine Mile (Coxevac, CEVA Inc.)
Francisella spp. - *F. tularensis* strain LVS (NCTC 10857)
Piroplasms - *Babesia canis* from dog blood

References

Regnery RL, Spruill CL, Plikaytis BD. Genotypic identification of rickettsiae and estimation of intraspecies sequence divergence for portions of two rickettsial genes. J Bacteriol. 1991;173:1576–1589.

Heylen D, Tijsse E, Fonville M, Matthysen E, Sprong H. Transmission dynamics of *Borrelia burgdorferi* s.l. in a bird tick community. Environ Microbiol. 2013;15:663–673.

Hovius JWR, de Wever B, Sohne M, Brouwer MC, Coumou J, Wagemakers A, Oei A, Knol H, Narasimhan S, Hodiamont CJ, Jahfari S, Pals ST, Horlings HM, Fikrig E, Sprong H, van Oers MHJ. A case of meningoencephalitis by the relapsing fever spirochaete *Borrelia miyamotoi* in Europe. Lancet 2013;382:658.

Szekeres S, Coipan EC, Rigó K, Majoros G, Jahfari S, Sprong H, Földvári G. Eco-epidemiology of *Borrelia miyamotoi* and Lyme borreliosis spirochetes in a popular hunting and recreational forest area in Hungary. Parasit Vectors. 2015;8:309.

Casati S, Sager H, Gern L, Piffaretti JC. Presence of potentially pathogenic *Babesia* sp. for human in *Ixodes ricinus* in Switzerland. Ann Agric Environ Med. 2006;13:65–70.

Loftis AD, Reeves WK, Szumlas DE, Abbassy MM, Helmy IM, Moriarity JR, Dasc GA. Rickettsial agents in Egyptian ticks collected from domestic animals. Exp Appl Acarol. 2006;40:67–81.

Versage JL, Severin DDM, Chu MC, Petersen JM. Development of multitarget real-time TaqMan PCR assay for enhanced detection of *Francisella tularensis* in complex specimens. J Clin Microbiol. 2003;41:5492–5499.
