# Supplementary figures and images for: Anaplasmataceae closely related to Ehrlichia chaffeensis and Neorickettsia helminthoeca from birds in Central Europe, Hungary
Source: Antonie Van Leeuwenhoek. 2020 Apr 21;113(7):1067–73. doi: 10.1007/s10482-020-01415-4 (PMC7272389; doi:10.1007/s10482-020-01415-4)

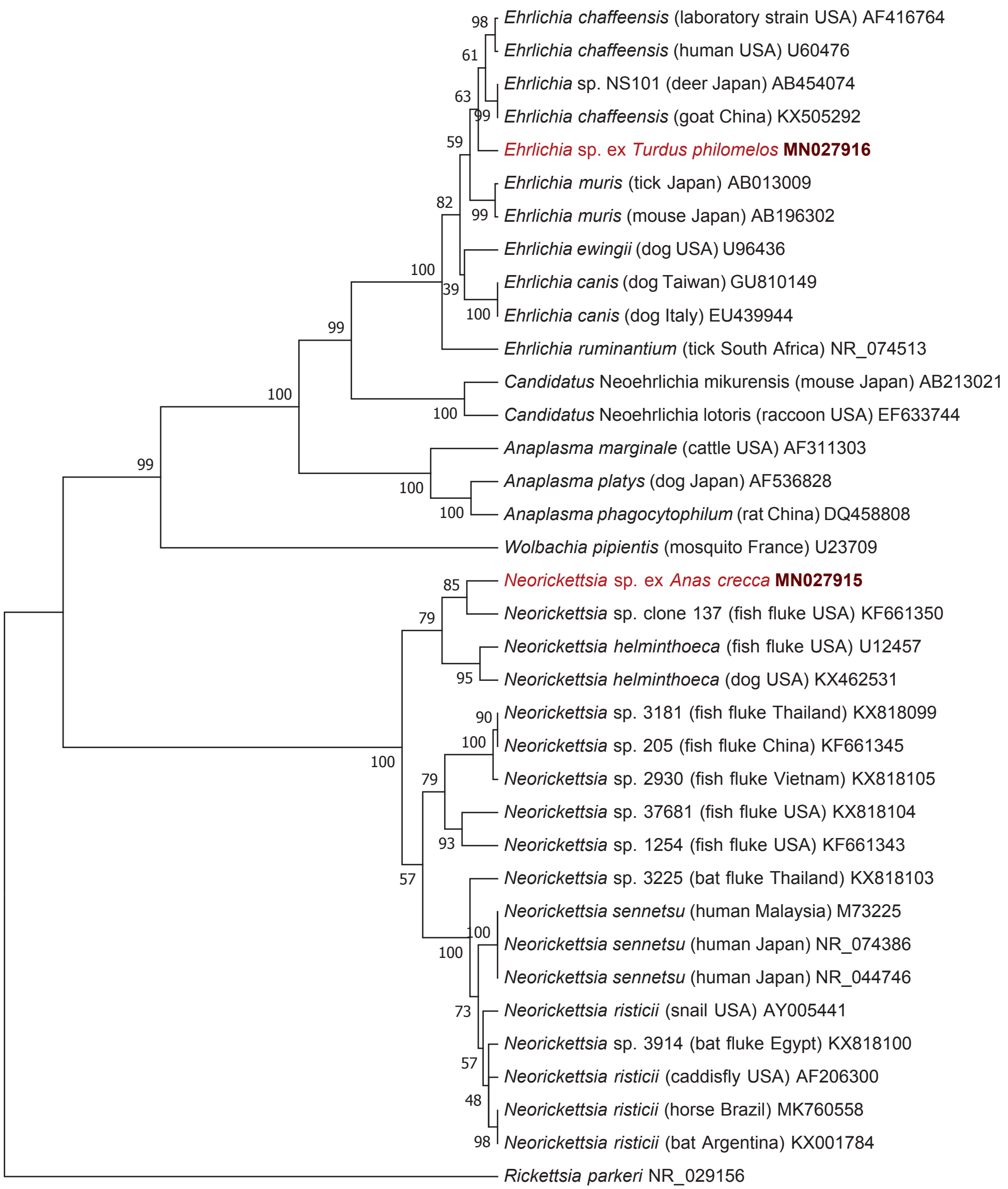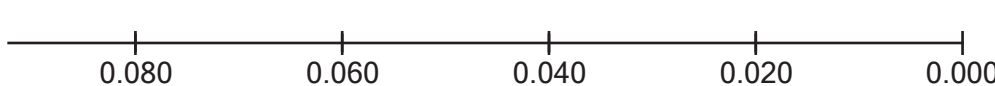

Supplement: Supplementary file 3 — Supplementary file3 (PDF 326 kb) [file 10482_2020_1415_MOESM3_ESM.pdf]

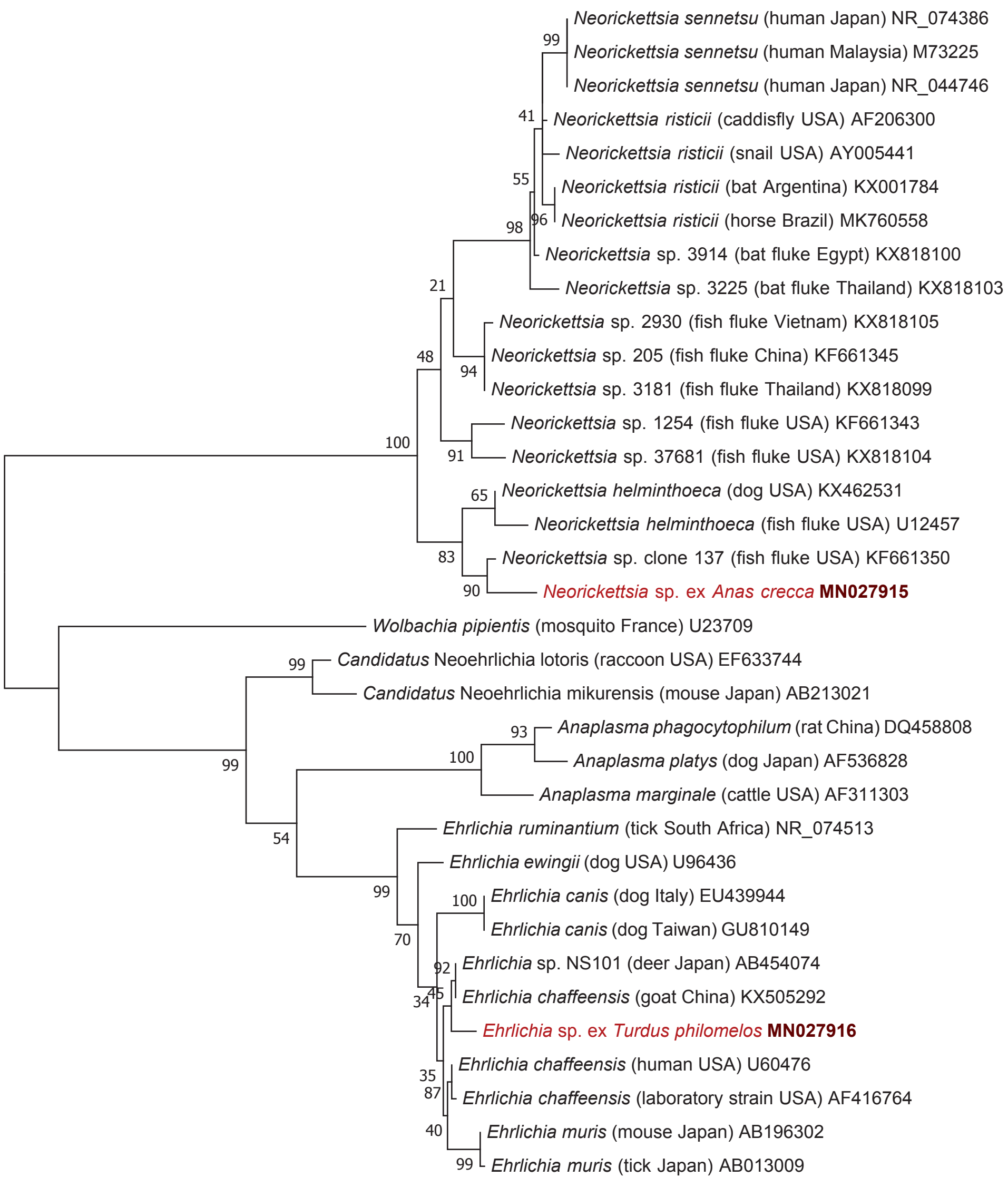

0,020

Supplement: Supplementary file 4 — Supplementary file4 (PDF 781 kb) [file 10482_2020_1415_MOESM4_ESM.pdf]
